# Lack of *Kcnn4* improves mucociliary clearance in muco-obstructive lung disease

**DOI:** 10.1172/jci.insight.140076

**Published:** 2020-08-20

**Authors:** Génesis Vega, Anita Guequén, Amber R. Philp, Ambra Gianotti, Llilian Arzola, Manuel Villalón, Olga Zegarra-Moran, Luis J.V. Galietta, Marcus A. Mall, Carlos A. Flores

**Affiliations:** 1 Centro de Estudios Científicos, Valdivia, Chile.; 2 Universidad Austral de Chile, Valdivia, Chile.; 3 Istituto Giannina Gaslini, Genoa, Italy.; 4 Departamento de Fisiología, Pontificia Universidad Católica de Chile, Santiago, Chile.; 5 Telethon Institute of Genetics and Medicine, Pozzuoli, Italy.; 6 Department of Translational Medical Sciences, University of Naples Federico II, Naples, Italy.; 7 Department of Pediatric Pulmonology, Immunology and Critical Care Medicine, Charité — Universitätsmedizin Berlin, Berlin, Germany.; 8 Berlin Institute of Health, Berlin, Germany.; 9 German Center for Lung Research, Berlin, Germany.

**Keywords:** Inflammation, Pulmonology, Epithelial transport of ions and water, Potassium channels

## Abstract

Airway mucociliary clearance (MCC) is the main mechanism of lung defense keeping airways free of infection and mucus obstruction. Airway surface liquid volume, ciliary beating, and mucus are central for proper MCC and critically regulated by sodium absorption and anion secretion. Impaired MCC is a key feature of muco-obstructive diseases. The calcium-activated potassium channel KCa.3.1, encoded by *Kcnn4*, participates in ion secretion, and studies showed that its activation increases Na^+^ absorption in airway epithelia, suggesting that KCa3.1-induced hyperpolarization was sufficient to drive Na^+^ absorption. However, its role in airway epithelium is not fully understood. We aimed to elucidate the role of KCa3.1 in MCC using a genetically engineered mouse. KCa3.1 inhibition reduced Na^+^ absorption in mouse and human airway epithelium. Furthermore, the genetic deletion of *Kcnn4* enhanced cilia beating frequency and MCC ex vivo and in vivo. *Kcnn4* silencing in the *Scnn1b*-transgenic mouse (*Scnn1b*^tg/+^), a model of muco-obstructive lung disease triggered by increased epithelial Na^+^ absorption, improved MCC, reduced Na^+^ absorption, and did not change the amount of mucus but did reduce mucus adhesion, neutrophil infiltration, and emphysema. Our data support that KCa3.1 inhibition attenuated muco-obstructive disease in the *Scnn1b*^tg/+^ mice. K^+^ channel modulation may be a therapeutic strategy to treat muco-obstructive lung diseases.

## Abstract 2

Silencing the calcium-activated potassium channel KCa.3.1 improves mucociliary clearance in muco-obstructive lung disease by decreasing sodium absorption in the airways.

## Introduction

Mucociliary clearance (MCC) is a key process, which sustains airway innate defense, and its proper function relies on coordinate regulation of epithelial ion and fluid transport, mucus homeostasis, and ciliary beating (1, 2). Disruption of any of these epithelial functions reduces airway clearance capacity, as observed in asthma, chronic obstructive pulmonary disease (COPD), idiopathic pulmonary fibrosis or primary ciliary dyskinesia, all lung diseases characterized by mucus accumulation, airway obstruction, infections, and progressive bronchiectasis (3–5). Cystic fibrosis (CF), the most common genetic disorder in humans, is the “flagship” of all muco-obstructive diseases. Mutations in the *CFTR* gene impede anion transport through the CFTR protein, which reduce lung function dramatically (6). The absence of chloride secretion into the airway lumen dehydrates the airway surface liquid (ASL) and impairs cilia movement, whereas absent HCO_3_^–^ secretion (that can be secondary to Cl^–^/HCO_3_^–^ exchange or directly through CFTR) affects mucins deployment, making it sticky and hard to transport. Reduced HCO_3_^–^ secretion also produces acidic pH, which in turn decreases activity of ASL antimicrobial molecules, favoring infection settlement (7).

Although there is an ongoing discussion regarding the existence of increased Na^+^ absorption in CF tissues (8–11), its contribution to airway malfunction is tangible in a transgenic animal model with epithelial Na^+^ channel (ENaC) hyperactivity in the airways, which induce a muco-obstructive and inflammatory lung disease phenotype that shares key features with CF and COPD (12, 13). This observation goes in hand with the attempts to use ENaC channel blockers as a therapy for patients with CF that have shown improvements in mucus clearance but of short duration (14–16) or with occurrence of renal side effects (17). Genetic silencing using siRNA-based strategies directed against ENaC produce long-lasting increase in ASL and cilia beating frequency of human bronchial epithelial cells (HBECs) from patients with CF (18, 19), indicating again that reduction of Na^+^ absorption is of potential benefit in CF and hence other muco-obstructive disorders.

The mechanism for Na^+^ absorption, as described by Koefoed-Johnsen and Ussing in the late 1950s, requires basolateral K^+^ channel activity to sustain the electrical membrane gradient (20), and so Na^+^ absorption in the airways can be hypothetically regulated by basolateral K^+^ channel function. Tens of K^+^ channels are expressed in the airway epithelium, but in most cases their role in ion transport mechanisms of the lower airway epithelium has not been tested (21).

In this study, we determined the role of the KCa3.1 channel using inhibitors and *Kcnn4* silencing in ion transport and MCC. Using an animal model of CF/COPD–like muco-obstructive lung disease, we generated a double mutant animal to test the hypothesis that *Kcnn4* silencing ameliorates airway disease.

## Results

### Na^+^ absorption and calcium-activated anion secretion is dependent on KCa3.1 activity in mouse and human airway epithelium.

**Figure 1:**
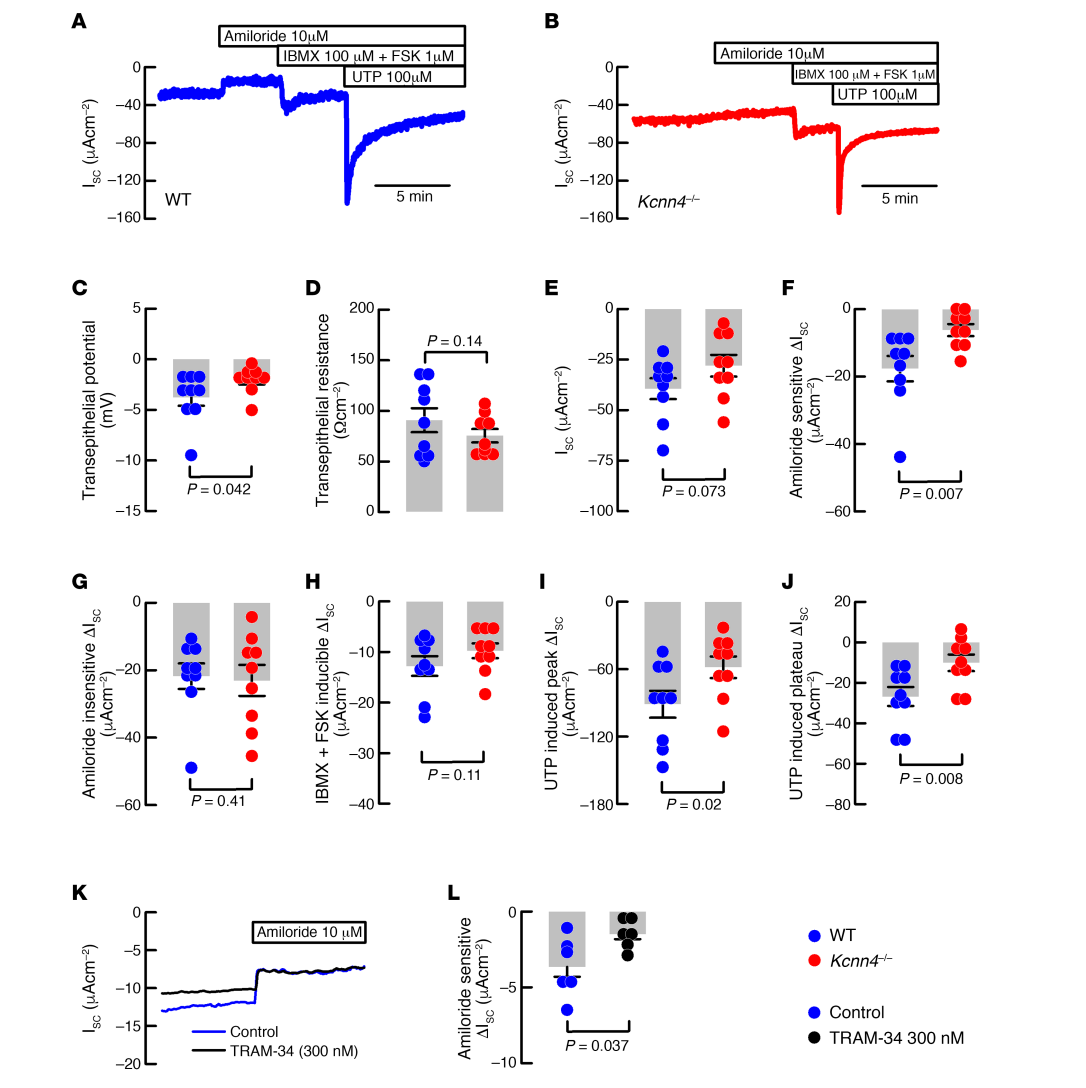
KCa3.1 participates in sodium absorption and anion secretion of mouse and human epithelium. Representative short-circuit current traces of (**A**) WT and (**B**) *Kcnn4*^–/–^ mouse tracheae used to calculate the following: (**C**) V_te_, (**D**) R_te_, (**E**) I_SC_, (**F**) Amiloride-sensitive current, (**G**) amiloride-insensitive current, (**H**) cAMP-induced anion current and Ca^+2^-activated anion current at (**I**) peak or (**J**) plateau phases; *n* = 9 for each group. (**K**) Representative Ussing chamber recordings of HBECs incubated with TRAM-34 and controls showing amiloride addition. (**L**) Summary of TRAM-34 effect on amiloride-sensitive Na^+^ absorption; *n* = 6 different cell cultures for each condition. Statistical differences were calculated using rank-sum test. Detailed values including data for *Kcne3*^–/–^ are included in Table 1.

First, we aimed to determine if the genetic deletion of *Kcnn4* affected electrogenic ion transport in the airway epithelium of mouse trachea. Representative traces of freshly excised WT and *Kcnn4*^–/–^ tracheal tissues are presented in Figure 1, A and B. We observed that the transepithelial potential (V_te_) was significantly reduced in the *Kcnn4*^–/–^ trachea when compared with WT trachea (Figure 1C). We observed no changes in transepithelial resistance (R_te_) (Figure 1D) or basal short-circuit current (I_sc_) (Figure 1E). The amiloride-sensitive current was significantly reduced in the *Kcnn4*^–/–^ tissues (Figure 1F). Both the amiloride-insensitive (Figure 1G) and the cAMP-induced anion secretion (Figure 1H) remained unaffected by *Kcnn4* silencing. The peak response of Ca^2+^-activated anion secretion induced by uridine-5′-triphosphate (UTP) (Figure 1I) and the plateau phase (5 minutes after UTP addition; Figure 1J) were reduced. Bioelectrical parameters are summarized in Table 1. We also tested if the silencing of another K^+^ channel, the KCNQ1/KCNE3, that is also important for anion secretion affected Na^+^ absorption using *Kcne3*^–/–^ tracheae, but we observed no changes (Table 1).

To determine if KCa3.1 inhibition mimics the observations in mouse trachea, we next tested known inhibitors of KCa3.1 channels, clotrimazol and TRAM-34, in HBEC monolayers mounted in Ussing chambers. Clotrimazol (30 μM) did not affect amiloride-sensitive Na^+^ absorption but produced a significant reduction in both cAMP- and Ca^2+^-activated anion secretion (Supplemental Figure 1, A–C; supplemental material available online with this article; https://doi.org/10.1172/jci.insight.140076DS1). TRAM-34 produced a significant reduction of the amiloride-sensitive current when used at 300 nM (Figure 1, K and L), with no effect on cAMP- or Ca^2+^-activated anion secretion (Supplemental Figure 1, B and C). When TRAM-34 was used at 100 nM, there was no effect on any of the measured parameters (Supplemental Figure 1, A–C).

To dispense with the idea that reduction in the amiloride-sensitive current is produced by a reduced expression of ENaC subunits or by a reduction in the epithelial protease and ENaC activator prostasin (*Prss8*), we performed qRT-PCR analysis and demonstrated that all ENaC subunits and *Prss8* gene expression remained unaltered after *Kcnn4* silencing (Supplemental Figure 2).

### KCa3.1 inhibition increases ciliary beating frequency and clearance in mouse airways.

**Figure 2:**
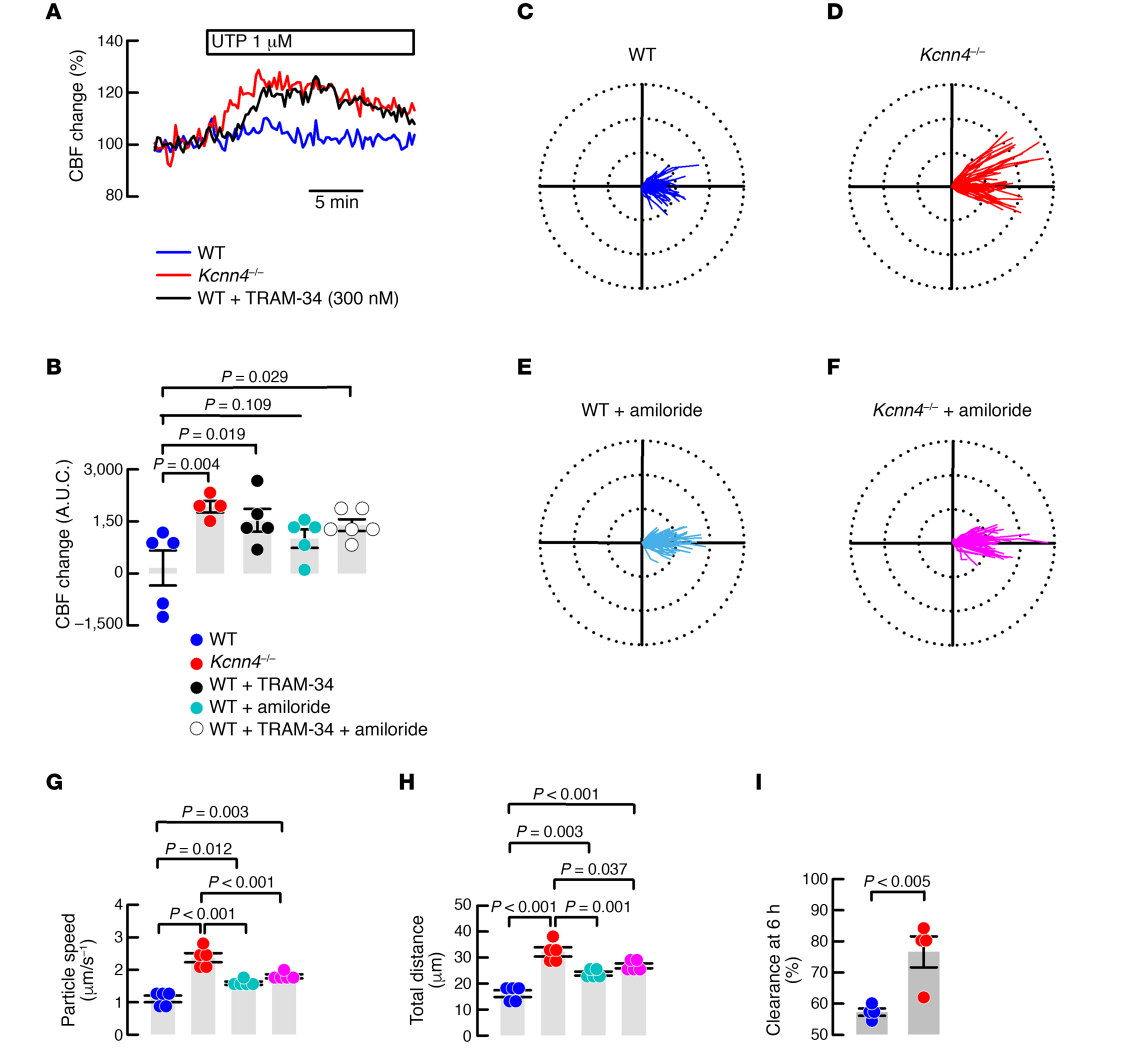
Mucociliary clearance is enhanced after KCa3.1 inhibition in the mouse. (**A**) CBF changes in *Kcnn4*^–/–^ or WT cells treated with TRAM-34 (100 nM) after UTP stimulation of tracheal epithelial cell explants. (**B**) Quantification of the area under the curve of CBF recordings including WT explants treated with amiloride (10 μM) and/or TRAM-34. Differences were calculated using ANOVA on ranks; *n* = 5, 4, 5, 5 and 6, respectively. Polar plots (75 μm radius) of the trajectory of beads placed in (**C**) WT (80 beads), (**D**) *Kcnn4*^–/–^ (68 beads), (**E**) WT plus 10 μM amiloride (84 beads), and (**F**) *Kcnn4*^–/–^ plus 10 μM amiloride (81 beads) tracheae, corresponding to 5 different experiments for each group. Calculated speed of particles (**G**) and total distance (**H**) from the experiments shown in **C–F**. Differences were calculated using ANOVA on ranks. (**I**) In vivo lung clearance of fluorescently labeled OVA in WT (*n* = 4) and *Kcnn4*^–/–^ (*n* = 4) mice. Difference was calculated using rank-sum test. CBF, ciliary beating frequency; UTP, uridine-5′-triphosphate.

To test if reduced Na^+^ absorption affected MCC, we studied changes in UTP-induced ciliary beating frequency (CBF), as determined in *Kcnn4*^–/–^ or WT mouse airway cells cultures incubated with TRAM-34 or/and amiloride. As observed in Figure 2A, genetic silencing or TRAM-34 increased UTP-induced CBF. The summary in Figure 2B includes experiments with amiloride with or without addition of TRAM-34. Amiloride alone did not significantly increase CBF, except when combined with TRAM-34.

Since CBF experiments must be performed in submerged conditions, we tested the clearance of plastic beads in freshly excised mouse tracheae, whose mucosal side was exposed to air in a humidified chamber. Figure 2, C and D, shows polar plots for WT and *Kcnn4*^–/–^ tissues in which an increase in the distance covered by the beads is clearly seen. Detailed analysis of the movement of the beads showed that *Kcnn4* silencing induced a significant increase in the average speed (Figure 2G) and the total covered distance (Figure 2H). To evaluate if inhibition of Na^+^ absorption affected the speed and distance traveled by the plastic beads, we used amiloride (Figure 2E) and observed an increase in both parameters in WT tracheae (Figure 2, G and H), as previously demonstrated (22). Nevertheless, when amiloride was tested in the *Kcnn4*^–/–^ tracheae, a decrease in MCC parameters was observed compared with nontreated *Kcnn4*^–/–^ tracheae (Figure 2F), but still maintained higher MCC parameters than control WT tissues (Figure 2, G and H). Finally, we tested whole lung clearance in vivo, measuring the clearance of fluorescent-labeled OVA and found a higher clearance percentage in the *Kcnn4*^–/–^ animals correlating with the ex vivo observations and demonstrating that *Kcnn4* silencing increases MCC (Figure 2I).

### Genetic silencing of Kcnn4 reduces Na^+^absorption and increases MCC in the Scnn1b^tg/+^ mouse.

**Figure 3:**
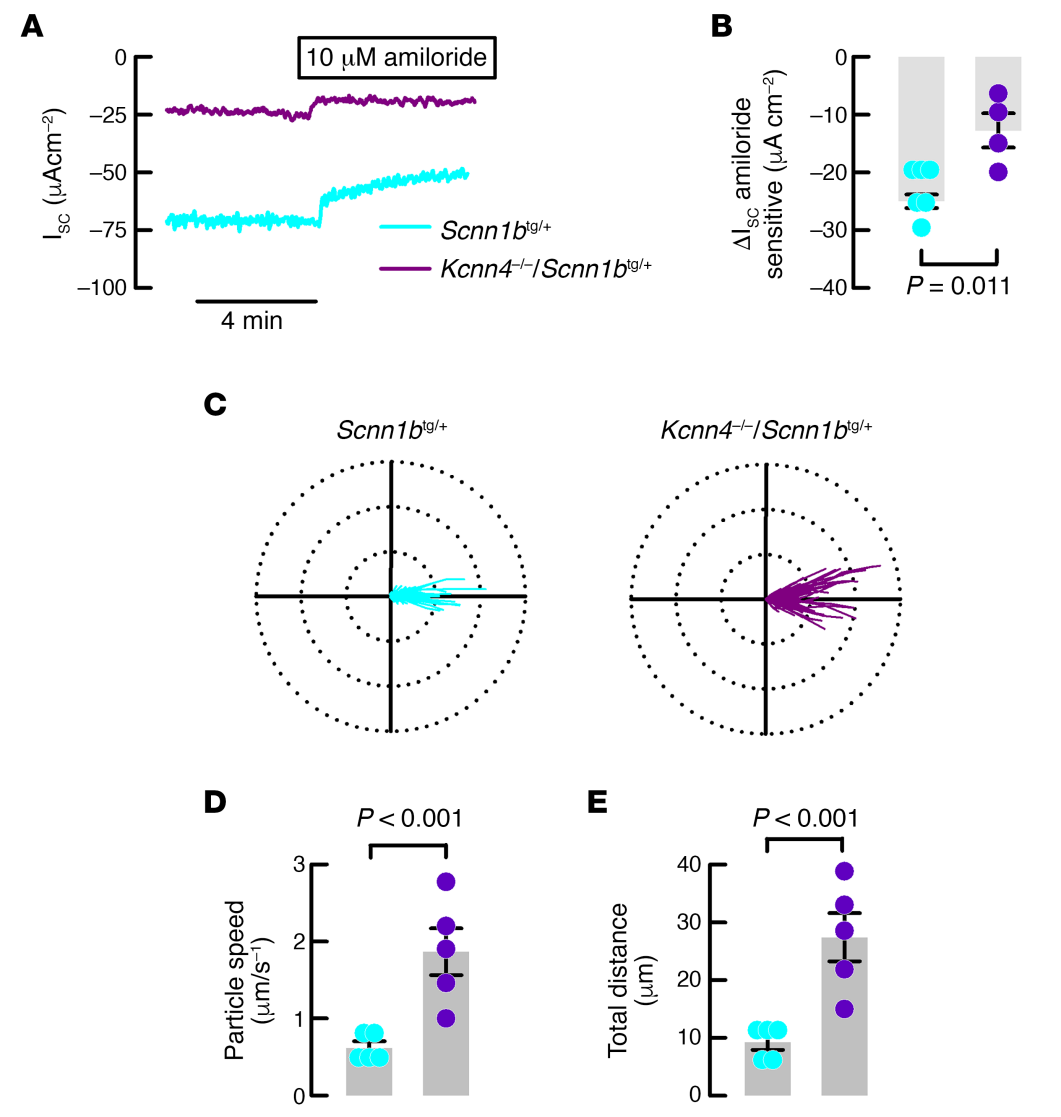
The genetic silencing of Kcnn4 reduces sodium absorption and increases MCC in the Scnn1b^tg/+^ mouse trachea. (**A**) Representative I_sc_ traces showing the extent of amiloride-sensitive currents in the *Scnn1b*^tg/+^ (*n* = 6) and double mutant (*n* = 4) mice tracheae. (**B**) Summary of amiloride-sensitive currents as shown in (**A**), *P* value calculated using ANOVA on ranks. Polar plots (75 μm radius) of the trajectory of beads placed in (**C**) *Scnn1b*^tg/+^ (222 beads) and double mutants (126 beads), from 5 different experiments each. Detailed electrical parameters are given in Table 1. Calculated (**D**) speed of particles and (**E**) total distance from the experiments shown in **C**. Differences were calculated using ANOVA on ranks. MCC, mucociliary clearance.

To test if the improved MCC observed in the *Kcnn4*^–/–^ mouse might be of potential benefit, we mated the *Kcnn4*^–/–^ animals with the *Scnn1b*^tg/+^ mouse that is affected by severe inflammatory and muco-obstructive airway disease. First, we studied if *Kcnn4* silencing induced changes in epithelial Na^+^ absorption and airway clearance. Ussing chamber experiments demonstrated that amiloride-sensitive Na^+^ absorption was significantly decreased in double mutants compared with tracheae from the *Scnn1b*^tg/+^ mouse (Figure 3, A and B), reaching values similar to those observed in the WTs (Table 1). The clearance of beads also showed increased values for speed and distances (Figure 3, C–E), indicating that *Kcnn4* silencing improved MCC in the muco-obstructive lung disease model.

### Genetic silencing of Kcnn4 prevents airway inflammation and lung damage in the Scnn1b^tg/+^ mouse.

**Figure 4:**
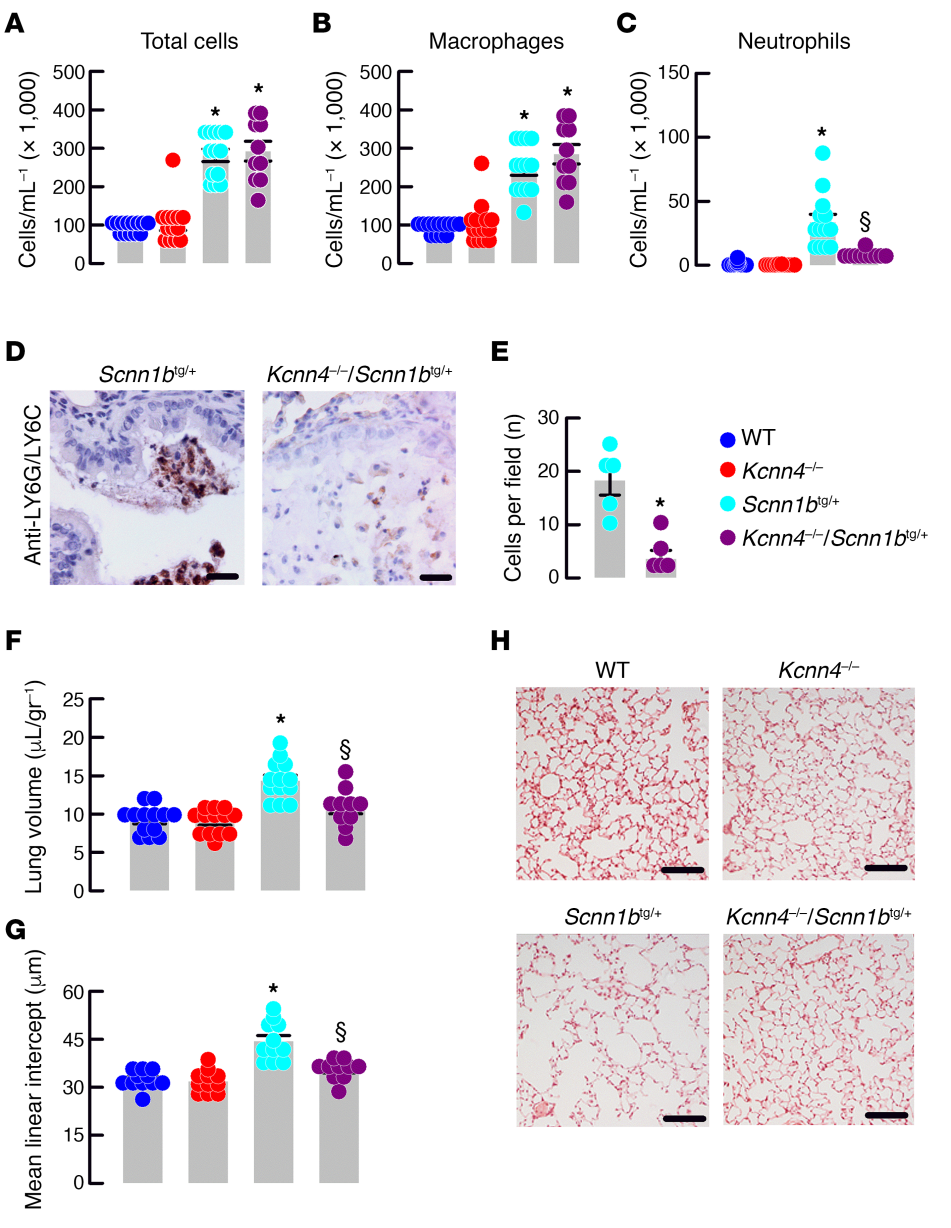
Genetic silencing of Kcnn4 reduces lung inflammatory disease in mice with muco-obstructive lung disease. Total cells (**A**) and macrophages (**B**) quantification in BALF. *P < 0.05 vs. WT and *Kcnn4*^–/–^. Neutrophils (**C**) quantification in BALF. *P < 0.05 vs. all other groups and § indicates the difference vs. *Scnn1b*^tg/+^. ANOVA on ranks; *n* = 13, 13, 13, and 10 for WT, *Kcnn4*^+/+^, *Scnn1b*^tg/+^, and double mutants, respectively. Representative images (*n* = 5 each group; scale bar: 20 μm) of LY6G/LY6C immunostaining in mucus plugs (**D**). Quantification of LY6G/LY6C-positive cells (**E**) in the *Scnn1b*^tg/+^ (*n* = 5) and double mutants (*n* = 5). *P < 0.05 calculated by rank-sum test. Mouse lung volume (**F**); *P < 0.05 vs. all other groups and § indicates the difference vs. *Scnn1b*^tg/+^; ANOVA on ranks; *n* = 13, 13, 13, and 10 for WT, *Kcnn4*^+/+^, *Scnn1b*^tg/+^, and double mutants, respectively. Mean linear intercept (**G**) calculated from images as shown in **H** (scale bar: 200 μm); *P < 0.05 vs. all other groups and § indicates the difference vs. *Scnn1b*^tg/+^; ANOVA on ranks; *n* = 13, 13, 13, and 10 for WT, *Kcnn4*^+/+^, *Scnn1b*^tg/+^, and double mutants, respectively.

After *Kcnn4* silencing lung edema in the *Scnn1b*^tg/+^ mouse was reduced to levels similar to the control animals (Figure 4A) and concomitantly, we observed less damage associated with emphysema (Figure 4, B and C). Analysis of bronchoalveolar lavage fluid (BALF) was performed to quantify the absolute numbers and relative distribution of immune cell types present in the airways of the animals. As previously observed (12), the *Scnn1b*^tg/+^ mouse showed increased amounts of total cells (Figure 4D), corresponding to macrophages (Figure 4E) and neutrophils (Figure 4F). The silencing of *Kcnn4* did not affect the number of total cells or macrophages but reduced the number of neutrophils in the *Scnn1b*^tg/+^ airways, an observation that was confirmed with staining and quantification of neutrophils in mucus plugs with LY6G/LY6C (Figure 4, G and H). The silencing of *Kcnn4* alone did not produce changes in the measured parameters (Figure 4).

**Figure 5:**
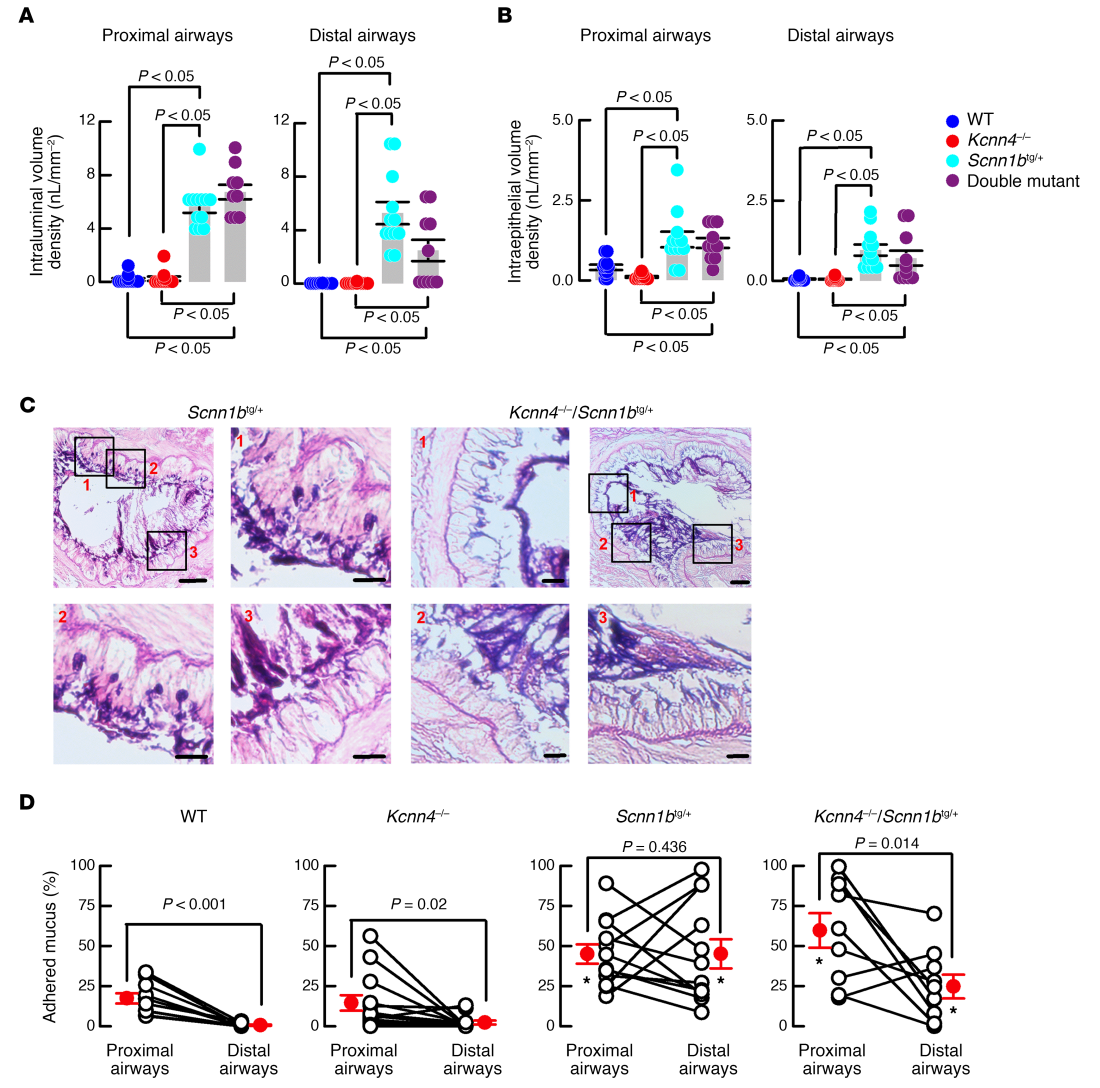
Genetic silencing of Kcnn4 improved mucus clearance in mouse airways. Intraluminal (**A**) and intracellular (**B**) mucus volume was determined in the proximal and distal airways. Differences were calculated with ANOVA on ranks; *n* = 12, 12, 12 and 9 animals for WT, *Kcnn4*^+/+^, *Scnn1b*^tg/+^, and double mutants, respectively. Representative images of mucus attachment to the epithelial surface for *Scnn1b*^tg/+^ (*n* = 15) and double mutants (*n* = 13) (**C**). Selected areas of main images (scale bar: 50 μm) are noted as 1–3 in red letters, and are shown amplified separately (scale bar: 20 μm). Summary of the percentage of epithelium surface covered my mucus in proximal and distal airways (**D**). Only paired samples from the same animal were included; *n* = 13, 16, 15, and 13 for WT, *Kcnn4*^+/+^, *Scnn1b*^tg/+^, and double mutants, respectively. *P < 0.05 vs. WT and *Kcnn4*^–/–^ ANOVA on ranks. The *P* values for each proximal vs. distal airways comparison were calculated by rank-sum test.

To test if the improvement in lung disease correlates with a decrease in mucostasis, we analyzed lung histological samples corresponding to proximal bronchi and distal bronchi stained with periodic acid–Schiff (PAS). Intraluminal and intraepithelial mucus volume were increased in the *Scnn1b*^tg/+^ mouse compared with WTs and *Kcnn4*^–/–^ in both proximal and distal airways (Figure 5, A and B). After *Kcnn4* silencing, values were not significantly different from those observed in the *Scnn1b*^tg/+^ mouse, but we observed a trend toward reduced intraluminal mucus obstruction in the distal airways of double mutants *Kcnn4*^–/–^/*Scnn1b*^tg/+^ mice that was not statistically significant based on the number of mice available for these studies (Figure 5A). A closer examination of the tissues led us to the observation that although mucus in the *Scnn1b*^tg/+^ mouse mostly adhered to the epithelial wall, this was not the case in the double mutant airways (Figure 5C). Quantification of the epithelial airway surface in contact with mucus demonstrated that there was a gradient of mucus adhesion to the surface of the epithelium (proximal to distal airway gradient) in the WT animals (17.4% ± 3% and 0.4 ± 0.1% of epithelial surface attached to mucus; *P* < 0.001) that was maintained in the *Kcnn4*^–/–^ tissues (14.6% ± 4.8% and 2.3% ± 1.2%; *P* = 0.02; Figure 5D). Analysis of the *Scnn1b*^tg/+^ tissues showed a complete loss of the proximal to distal airway gradient, as both values were similar and higher in both proximal and distal airways (45.0% ± 6% and 45.1% ± 9.1%; *P* > 0.4). Finally, a recovery of the proximal to distal airway gradient was observed after *Kcnn4* silencing (59.7% ± 10.8% and 24.7% ± 7.4%; *P* = 0.014).

## Discussion

We identified KCa3.1 as a K^+^ channel responsible for energizing the cell electrical gradient for Na^+^ absorption in human and mouse airway epithelial cells. As predicted from previous observations, reduced Na^+^ absorption might enhance MCC function in the airways of the *Kcnn4*^–/–^ mouse; therefore, we tested the potential of *Kcnn4* silencing in the amelioration of airway muco-obstructive disease in the *Scnn1b*^tg/+^ mouse model. *Kcnn4* silencing successfully decreased inflammatory lung damage, neutrophilic inflammation, and improved MCC in the *Scnn1b*^tg/+^ animals. We found that *Kcnn4* silencing also affected anion secretion; however, no signs of muco-obstructive disease were found in this model.

Early observations in patients affected with pseudohypoaldosteronism (due to ENaC-inactivating mutations) indicated that ENaC activity was inversely related to ASL volume and MCC efficiency (23), suggesting that ENaC might be a druggable target to manage muco-obstructive lung disease. Some rate of success is obtained using ENaC blockers, such as amiloride and derivatives, but short duration or side effects hampered further advances in the development of therapies and so new approaches are necessary (24). As described in the 1950s, the Na^+^ absorption mechanism relies on the activity of basolateral K^+^ channels (20). Acting in concert with the Na^+^ pump, highly selective basolateral channels allow the extrusion of K^+^ that, if retained, depolarizes the membrane potential, hampering Na^+^ absorption. This is the case in the kidney and intestine where inhibition of basolateral K^+^ channels reduced Na^+^ absorption and Na^+^-coupled metabolite uptake (25–27), further strengthening the role of K^+^ channels on epithelial Na^+^ homeostasis. We envisioned that Na^+^ absorption in the airways can be controlled through basolateral K^+^ channels, but their role in electrolyte transport has not been profusely studied in this tissue (21). With the exceptions of Kir7.1, KCNE3, and KCa3.1, no other K^+^ channels or subunits are located in the basolateral membrane of lower airway epithelium (28–30). Experiments with ion channel inhibitors demonstrate that KCNQ1/KCNE3, using chromanol-293B, and KCa3.1, using clotrimazole, participate in the mechanism of anion secretion of the airways, and experiments in the *Kcne3*^–/–^ mouse confirm its role, but no effect on Na^+^ absorption is observed (28, 31, 32). Our results confirmed these observations, as when using clotrimazole a decrease in anion secretion was observed, but there was no effect in Na^+^ absorption in human cells, similar to what we observed in tracheae from the *Kcne3*^–/–^ animals. When using the KCa3.1 opener 1-EBIO, we observe an increase in Na^+^ absorption in HBECs, supporting a role for KCa3.1 as a regulator of Na^+^ absorption in these cells (33), a result comparable with our observations using TRAM-34 in HBECs and tracheae from the *Kcnn4*^–/–^ mouse where decreased Na^+^ absorption was recorded. The *Kcnj13*^–/–^ mouse bears a lethal phenotype at perinatal age and is affected by severe tracheomalacia, but it is not known if Kir7.1 participates in the anion secretion mechanism and if it relates to the observed phenotype (34).

Epithelial anion secretion in the airways is also affected after K^+^ channel silencing that a priori, and in the scenario of a muco-obstructive disease, it is unwanted. Inhibition of both KCa3.1 and KCNQ1/KCNE3 produces a significant reduction of Ca^2+^- and cAMP-induced anion secretion, respectively (31, 32), but no muco-obstructive phenotype is observed in the *Kcne3*^–/–^ airways (28), nor was it observed in the *Kcnn4*^–/–^ airways in our results. Such reductions in anion secretion are also innocuous in the intestine. For example, *Kcnn4*, *Kcnq1*, *Kcne3,* or *Kcnk5* silencing does not produce obstructions or CF-like phenotypes in the intestine of the animals, as expected from genes coding for K^+^ channels with tested roles in epithelial anion secretion (26, 28, 35, 36). Even though, we do not have a straight answer for the lack of the muco-obstructive phenotype when *Kcnn4* is silenced, it is important to consider that the inhibition of anion secretion in this model is not complete; KCNQ1/KCNE3 activity and basolateral electroneutral-anion influx can still support anion secretion at a sufficient level to maintain MCC function.

But is inhibiting KCa3.1 better than inhibiting ENaC? Besides the use of ENaC blockers and other inactivating strategies already discussed, animal models demonstrate that silencing of genes coding for ENaC subunits is lethal, and, in particular, *Scnn1a* silencing produces lethality due to defective liquid clearance from the neonatal lung (37, 38). Nevertheless, we agree with the idea that the key seems to be the dosage of ENaC inhibition, as the heterozygous mutant mice for *Scnn1a* do not show lethality, and residual ENaC activity in *Scnn1b*^–/–^ and *Scnn1g*^–/–^ is sufficient to overcome the neonatal lung phenotype (38–40). Our results show that, after *Kcnn4* silencing, approximately 35% of amiloride-sensitive Na^+^ absorption was still active in mouse trachea, suggesting that the remaining current is sufficient to maintain proper MCC function.

Analysis of the genetic silencing of *Kcnn4* in the *Scnn1b*^tg/+^ animal demonstrated reduced infiltration of neutrophils and decreased emphysemic damage of the respiratory epithelium. The reduction in neutrophils might be explained by a direct effect on the neutrophil’s chemotaxis capacity that is reduced after *Kcnn4* silencing (41), but additionally, the reduction of chemoattractants released might influence the arrival of inflammatory cells to the airways (42, 43). We also observed that a gradient of adhered mucus to the epithelium normally occurred, as mucus covers a larger surface in the proximal bronchi than in the terminal. This gradient is disrupted in the *Scnn1b*^tg/+^ tissues, where similar values for mucus attached to the epithelial surface are observed in proximal and distal bronchi. Although we observed a tendency for mucus reduction in the distal airways of double mutants, it was accompanied by a recovery in the gradient of mucus adherence to the epithelial surface, supporting the idea that the mucus hypersecretory state per se is not enough to provoke muco-obstructive disease. For example, animals that overexpress MUC5AC or MUC5B in the lungs do not produce airway obstructions or inflammation, even when in some cases the amount of mucus protein is almost 20 times higher than that in control animals, thus reaffirming the idea that under normal fluid transport the amount of mucus is not an issue (44, 45). Mucus oversecretion must then be accompanied by airway dehydration and inflammatory signaling to complete the muco-obstructive pathology. Therefore, we conclude that the observed reduction of inflammation and the increased MCC after *Kcnn4* silencing prevents the muco-obstructive pathology in the double mutant animals irrespective of mucus quantity. Even when mucus hypersecretion is at the center stage in airway muco-obstructive diseases, animal models demonstrate that impeding mucin expression is detrimental for animal well-being, because mucus is fundamental for lung health (45). Studying the same *Scnn1b*^tg/+^ mouse model with additional silencing of *Muc5ac* or *Muc5b*, increased inflammation or no improvements at all in airway disease is found (46). Our results agree with the idea that complete mucus reduction is not recommendable, but interventions that boost MCC, improve mucus quality, and/or reduce adhesion of mucus to the epithelium are in the road to limit the seriousness of the disease.

KCa3.1 possesses a high affinity for Ca^2+^, with EC_50_ values of 95–160 nM for the human and mouse channels, respectively (47, 48). Free calcium in the cytoplasm of airway epithelial cells is typically 100 nM (49, 50), but ciliated cells bear a higher concentration at baseline than club cells (51). Another important functional consideration is that ciliated cells respond, increasing Ca^2+^ from 100 nM up to 400 nM when stimulated with a low ATP concentration (0.1–1 μM) (52), close to what is normally found in the sputum and ASL from human donors (53–55). All this evidence argues in favor of the idea that KCa3.1 channels are active during normal conditions in certain cells of the airway epithelium, but further immunolocalization studies are necessary to unveil the exact cell distribution, subcellular localization, and protein interactions of KCa3.1 channels in the airways.

Even when decreased Na^+^ absorption after *Kcnn4* silencing fits with expected improvements in MCC, we have to address the fact that some of our results indicate that KCa3.1 functions go beyond epithelial fluid secretion and absorption. Increased CBF after KCa3.1 blockade occurs in submerged conditions, discarding a direct link to increased ASL. The hyperpolarization produced by the opening of K_ATP_ channels in the apical membrane increases the speed CBF (56); in a similar fashion, KCa3.1 inhibition might have a hyperpolarizing effect in the apical membrane (by decreasing Na^+^ absorption) boosting CBF. Our results also indicate that KCa3.1 inhibition had a greater effect on CBF than ENaC inhibition. It might be possible that the reduction of anion secretion also favored apical membrane hyperpolarization, a hypothesis that is supported by the more positive V_te_ recorded in the *Kcnn4*^–/–^ tissue that might be due to Na^+^ accumulation in the mucosa and reduced anion secretion. Besides that, KCa3.1 inhibition has been widely proven by different groups to prevent or ameliorate several inflammatory events, including those occurring in the airways (57–59). Since our model is a systemic null animal, other cells might also participate in the reduction of inflammatory damage after *Kcnn4* silencing. Such is the case of neutrophils, whose number is reduced in the *Kcnn4*^–/–^ lungs affected by acute lung injury (41). Similar results are obtained in the CF *Cftr*^ΔF508/ΔF508^ mouse, which after *Kcnn4* silencing, shows almost completely reduced lethality associated with intestinal obstructions, but this is not accompanied by improvements in intestinal anion secretion (42). Finally, the use of KCa3.1 inhibitors must be taken with caution because off-targets are reported (60–62), and in our case, the use of 2 different inhibitors (clotrimazol and TRAM-34) resulted in noncomparable results in Ussing chamber experiments. Senicapoc, a new and more potent inhibitor of KCa3.1, has been tested in clinical trials for sickle cell anemia and is shown to be safe and well tolerated in patients, offering a faster option for treatment of muco-obstructive diseases in humans (63).

Although patients with CF have found therapies in molecules that help correct mutant CFTR malfunctions (64, 65), and new such molecules are being discovered (66), almost 10% of patients with CF are still left with no such treatment. The potential use of KCa3.1 inhibitors corresponds with what has been called a “mutation-agnostic” treatment that might be used specially in patients with CF that are not eligible for the new available therapies, and it also seems suitable for easing other muco-obstructive diseases affecting humans. The absence of severe phenotypes in the *Kcnn4*-null animal (35, 67–69) suggests the use of KCa3.1 inhibitors may be safe in human diseases.

## Methods

### Reagents.

Unless stated otherwise, all reagents (amiloride, forskolin, IBMX and clotrimazole) were obtained from MilliporeSigma, with the exception of TRAM-34, which was obtained from TOCRIS.

### Animals.

Mice were housed at the Centro de Estudios Científicos (CECs) animal facility. *Kcnn4*^–/–^ animal generation and their genotyping have been described previously (67). The *Scnn1b*^tg/+^ mouse was backcrossed on a C57BL/6J background as previously described (70). Animals were maintained in the specific pathogen–free mouse facility of CECs with access to food and water ad libitum. Six-week-old male or female mice were used, all backcrossed for at least 20 generations on a C57BL/6J background.

### Primary HBEC culture.

The procedures for isolation and culture of HBECs were described in detail in a previous study (71). Briefly, HBECs were cultured in flasks in a serum-free medium (LHC9/RPMI 1640). After 4–5 passages, cells were seeded at high density (500,000/cm^2^) on Snapwell 3801 porous inserts (Corning Costar). Twenty-four hours after seeding, the medium was switched to DMEM/F12 (1:1) plus 2% New Zealand fetal bovine serum (Thermo Fisher Scientific), hormones, and supplements. The medium was replaced daily on both sides of the permeable supports for up to 6–7 days (liquid-liquid culture). The apical medium was subsequently removed and the cells received nutrients only from the basolateral side (air-liquid culture [ALC]). This condition favored further differentiation of the epithelium. Cells were maintained under ALC for 2 weeks before the experiments were performed. Experiments testing TRAM-34 used cells grown on BMIX media and experiments testing clotrimazol used CBMIX, as previously described (71). Only paired experiments from the same cells were included in the analysis.

### Ussing chamber experiments.

Tracheae were placed in P2306B of 0.057 cm^2^ tissue holders and placed in Ussing chambers (Physiologic Instruments Inc.). Tissues were bathed with bicarbonate-buffered solution (pH 7.4) of the following composition (in mM): 120 NaCl, 25 NaHCO_3_, 3.3 KH_2_PO_4_, 0.8 K_2_HPO_4_, 1.2 MgCl_2_, 1.2 CaCl_2_, and 10 d-Glucose, gassed with 5% CO_2_ and 95% O_2_, and kept at 37°C. The transepithelial potential difference referred to the serosal side was measured using a VCC MC2 amplifier (Physiologic Instruments Inc.). The short-circuit currents (I_SC_) were calculated using Ohm’s law, as previously described (72). Differences (Δ I_SC_), were calculated from I_SC_ after minus before the addition of drugs. The experiments were performed in the presence of 10 μM amiloride in the apical side to block Na^+^ absorptive currents. The 1 μM forskolin plus 100 μM 3-isobutyl-1-methylxanthine (IBMX) cocktail was used to stimulate cAMP-dependent anion secretion, and 100 μM UTP to evoke Ca^2+^-activated anion secretion. For experiments on HBECs, Snapwell supports carrying differentiated bronchial epithelia were mounted in a vertical chamber resembling an Ussing system with internal fluid circulation. Both apical and basolateral hemichambers were filled with 5 mL of a Krebs bicarbonate solution containing (in mM): 126 NaCl, 0.38 KH2PO4, 2.13 K2HPO4, 1 MgSO4, 1 CaCl2, 24 NaHCO3, and 10 glucose. Both sides were continuously bubbled with a gas mixture containing 5% CO_2_ and 95% air and the temperature of the solution was kept at 37°C. The transepithelial voltage was short-circuited with a voltage clamp (EVC-4000, World Precision Instruments) connected to the apical and basolateral chambers via Ag/AgCl electrodes and agar bridges (1 M KCl in 1% agar). The offset between voltage electrodes and the fluid resistance were canceled before experiments. The short-circuit current was recorded with a PowerLab 4/25 (ADInstruments) analogical to digital converter connected to a computer.

### CBF.

Primary cultures of mouse airway epithelia were analyzed, as previously described (73). For all experiments, the central area on each ALI culture was imaged, and the digital image signal was routed from the camera directly into a digital image acquisition board (National Instruments) within a Dell XPS 710 workstation. Images were analyzed with virtual instrumentation software (Sissons-Ammons Video Analysis, National Instruments), which is highly customized to quantify CBF. All of the recordings in the present experiments were made at original magnification, ×630. Whole-field analysis was performed, with each point measured representing one cilium. For each sample, the reported frequencies represent the arithmetic means of these values. Changes in CBF are normalized to the basal rate and reported as a ratio of stimulated/basal.

### Plastic beads clearance and in vivo MCC.

Speed of polystyrene beads in trachea samples was determined with some modifications of the previously described method (74). Briefly, mice were deeply anesthetized via i.p. injection of 120 mg/kg ketamine and 16 mg/kg xylazine and exsanguinated, and the trachea was isolated and mounted using insect’s needles in an extra thick blot paper (Bio-Rad), which was perfused with Ringer’s solution (gassed with 95%/5% O_2_/CO_2_) at a rate of 1 ml/min, at 37°C, and maintained in a humidified chamber. Polystyrene black-dyed microspheres (diameter 6 μm, 2.6% solid-latex, Polybead, Polyscience Inc.) were washed and diluted with physiological solution (0.5% latex) and 4 μl of particle solution were added onto the bronchial edge of the trachea. Particle transport was visualized by images every 5 seconds (2–5 minutes sampling) of at least 3 different fields using a Motic camera (Moticam 5.0). Stored images were measured to determine the velocity of single particles using NIH ImageJ software and MCC was expressed in μm/sec. Allergen clearance from the whole lung was evaluated by the elimination of fluorescently labeled OVA (Alexa Fluor 647) after intratracheal instillation, as previously described (13). 1 mg/mL OVA was labeled with Alexa Fluor 647 with the microscale Protein labeling kit (Alexa Fluor 647 Microscale Protein Labelling Kit, Life Technologies). Intratracheal instillation of 2.5 μg OVA dissolved in 20 μl PBS was performed and held over 6 hours. To determine allergen clearance in the whole lung, mice were anesthetized via intraperitoneal injection with 10 mg/kg ketamine and 16 mg/kg xylazine. Lung was extracted after 6 hours. To determine the allergen clearance, lung was mixed with PBS and protease Inhibitor 1X (Roche). The lung was homogenized and fluorescence intensity was read in a microplate reader. Clearance was calculated using the following formula: %MCC allergen = [1–fluorescence intensity at 6 hr]/[fluorescence intensity at 0 hr] × 100. The fluorescence intensity at baseline (*t* = 0) (arbitrary units) should not interfere between treatments.

### Lung tissue isolation, BALF, and histological analysis.

Anesthetized mice were euthanized by exsanguination, and the chest cavity was opened to ligate the left main bronchus. A blunt needle (20 gauge) was inserted through a small incision in the upper trachea and tied in place with 3.0 silk. After ligation of the left main-stem bronchus, BAL was performed on the right lobes by instilling a volume of sterile PBS (137 mM NaCl, 2.7 mM KCl, 10 mM Na2HPO4, 2 mM KH2PO4) at room temperature determined by the following formula: [mouse weight (g) × 0.035 ml/2 = mL PBS instilled]

BAL was performed by gently injecting and retrieving the PBS volume 3 times. This procedure was carried out a second time with an equal volume of PBS and fractions were pooled. Return volume was consistently greater than 80% of the instilled volume. BAL cells were pelleted by centrifugation at 300 *g* for 5 minutes at 4°C and the cell-free supernatant (BALF) was collected and stored at −80°C with protease inhibitors. BAL cells were resuspended in 50 μL PBS; 10 μl were counted with a hemocytometer (diluted in 10 μl Trypan blue); and 40 μl were diluted in 160 μl and displaced in Cytospin slides (StatSpin Cytofuge 2), air dried, and stained with modified Giemsa for differential cell counts of at least 200 cells per slide. After BAL, the left and right lung were immersed in 10% neutral-buffered formalin to prevent the dislodging of airway luminal contents. The right lung was used to evaluate the mean linear intercept and the left lung to quantify mucus density and attachment using NIH ImageJ software.

### Lung histology and mucus morphometry.

Morphometric analyses of airway mucus obstruction were performed in noninflated, immersion-fixed left lungs. Lungs were removed through a median sternotomy, fixed in 4% buffered formalin, and embedded in paraffin. Left lung was sectioned transversally at the level of the proximal intrapulmonary main axial airway near the hilus (proximal airways), and at the distal intrapulmonary axial airway, at 1500 μm distal to the hilus (distal airways). To quantify secreted mucus, morphometric analysis of stained sections was carried out by determining mucus volume density using CellF software, as previously described (13). Briefly, the length of the basal membrane of the airway epithelium was measured by the interactive image measurement tool, and the Alcian blue–PAS staining–positive (AB-PAS–positive) surface area within this boundary was measured by phase analysis according to the automatic threshold settings of the software. The volume density of intraepithelial mucus, representing the volume of lumen mucus content per surface area of the mucus basal membrane (nl/mm^2^), was determined from the intraepithelial surface area of AB-PAS positive mucus and the length of the basal membrane of the airway epithelium. Luminal mucus content relative to the luminal area was determined in a similar fashion. Mucus attached to the epithelium was quantified with NIH ImageJ software. Using the interactive tool, polygon selections of the entire epithelial surface was surrounded to obtain the total perimeter. Then, epithelial surface in contact with the mucus was quantified and percentage determined. Formalin-fixed, paraffin-embedded lung sections cut at a thickness of 5 μm were hydrated and pretreated with 3% hydrogen peroxide in methanol followed by incubation for 1 hour with 1:5000 *InVivoPlus* anti-mouse Ly6G/Ly6C (Gr-1) (Bio X Cell). Further blocking with serum and developing of immune reaction was performed with VECTASTAIN ABC systems (Vector labs).

### mRNA isolation and cDNA synthesis from epithelial cells.

Mice were killed by cervical dislocation and tracheal tissues were immediately extracted; tracheae were incubated with Pronase 30 μM at 37°C for 30 minutes. Trachea was placed in DMEM 10 mM d-Glucose and epithelium was isolated by scraping with tweezers and further homogenized in 250 μL Trizol (TRIzol Reagent) and RNA isolated following the manufacturer’s instructions. The dried pellet of RNA was resuspended with 35 μL nuclease free water and stored at –80°C. DNA contamination was avoided using DNAse treatment. The concentration and integrity of the RNA were determined by spectrophotometry. Total RNA was reverse transcribed into cDNA using the Superscript III RTPCR System according to the manufacturer’s recommendations. cDNA synthesis was performed on 2 μg RNA. cDNA integrity was checked using specific primers to cyclophilin and 50 ng template cDNA was added to the reaction mixture. *Cyclophilin* (*Ppia*) amplification was performed starting with a 5-minute template denaturation step at 95°C, followed by 30 cycles of denaturation at 95°C for 30 seconds and combined primer annealing/extension at 55°C. The relative brightness intensity of ethidium bromide-stained bands resolved on a 1.5% agarose gel was evaluated.

### Real-time PCR.

Quantification of *Scnn1a*, *Scnn1b*, *Scnn1g*, and *Prss8* expression was performed using SYBR Green detection in a LightCycler PCR machine according to the manufacturer’s instructions. We determined the PCR efficiency of each individual assay by serially measuring 100 ng cDNA from a pool of epithelia in triplicate. Only CT values of less than 40 were used for calculation of the PCR efficiency. All PCRs displayed an efficiency between 96% and 100%. Amplifications were performed starting with a 3-minute template denaturation step at 94°C, followed by 45 cycles of denaturation at 94°C for 20 seconds and combined primer annealing/extension at the gene-specific primer temperature for 30 seconds. All samples were amplified in triplicate and the mean was obtained for further calculations. Relative fold changes in target gene expression were quantified by the previously reported ΔΔCT method (75). Briefly, CT values were obtained for individual samples using the Rotor-Gene 6000 software 1.7 (Corbett Life Science), where the targets and reference (Cyclophilin) had the same cDNA concentration. ΔCT was calculated by subtracting the CT (target – reference). Primers and annealing temperatures are provided in Table 2.

### Statistics.

All statistical analyses were performed using SigmaPlot V12.1. Rank sum test for paired groups and 1-way ANOVA for multiple comparisons were used as indicated on each figure or table. A *P* value of less than 0.05 was considered significant. Error bars represent mean ± SEM, and single experiments are included in figures.

### Study approval.

All experimental procedures in animals were approved by the CECs Institutional Animal Care and Use Committee (1151142-2015) and followed the relevant guidelines and regulations. The CECs animal facility is AAALAC accredited. The collection of bronchial epithelial cells to investigate the mechanisms of transepithelial ion transport were specifically approved by the ethics committee of the Istituto Giannina Gaslini following the guidelines of the Italian Ministry of Health (updated registration number: ANTECER, 042-09/07/2018). Each patient provided informed consent using a form that was also approved by the ethics committee.

## Author contributions

CAF conceived the study. Mouse electrophysiology and data analysis were performed by ARP and CAF. HBEC electrophysiology and data analysis were performed by A. Gianotti, OZM, and LJVG, and CAF CBF experiments and data analysis were performed by ARP, LA, and MV. MCC in vivo and ex vivo was performed by GV and A. Guequén. Data analysis was performed by A. Guequén and CAF. BALF leukograms and analysis were performed by GV. Neutrophil IHC was performed by GV. Analysis was performed by CAF. Histological analysis was performed by GV and CAF. Experimental design was performed by GV. A. Guequén, A. Gianotti, MAM, and CAF wrote the paper with help from GV and MAM. All authors revised and approved the final version.

## Supplementary Material

Supplemental data

## Figures and Tables

**Table 2:**
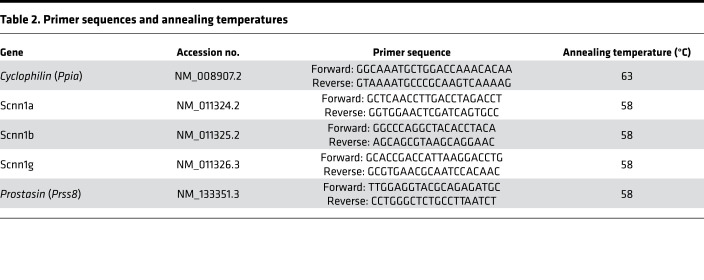
Primer sequences and annealing temperatures

**Table 1:**
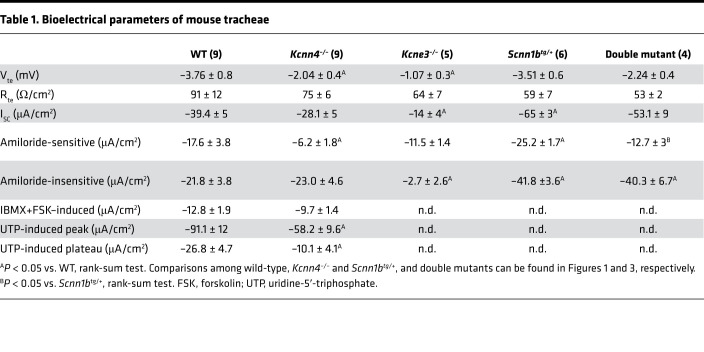
Bioelectrical parameters of mouse tracheae

## Version 1 08/20/2020

Electronic publication
