# Supplementary material for: Lack of Kcnn4 improves mucociliary clearance in muco-obstructive lung disease
Source: JCI Insight. 2020 Aug 20;5(16):e140076. doi: 10.1172/jci.insight.140076 (PMC7455130; doi:10.1172/jci.insight.140076)
Supplement: Supplemental data [file jciinsight-5-140076-s106.pdf]

SUPLEMENTAL FIGURE 1.

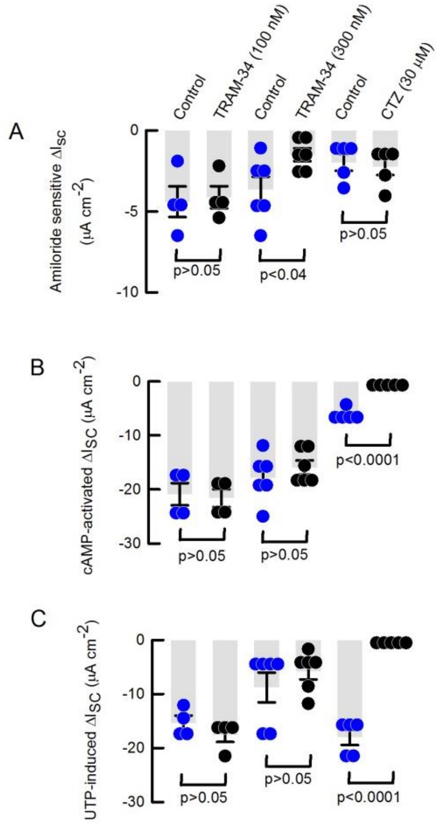

SUPLEMENTAL FIGURE 1. The KCa3.1 inhibitors TRAM-34 and clotrimazol (CTZ), have different effects on short-circuit currents measured in HBECs. (A) summarizes the amiloride-sensitive sodium-absorption. (B) cAMP and (C) Ca^{2+}-induced anion secretion was affected by CTZ only. All groups correspond to paired experiments. Calculation of the cAMP-induced ISC in the CTZ and corresponding controls were calculated using the $CFTR_{inh}172$ after cAMP; n=4 for TRAM-34 100 nM; n=6 for TRAM-34 300 nM and n=5 for CTZ 30 μM. Analysis performed using Rank sum test.

SUPLEMENTAL FIGURE 2.

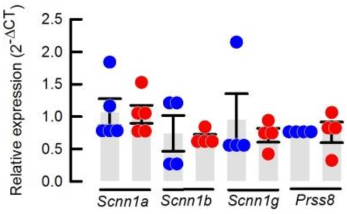

SUPLEMENTAL FIGURE 2. Silencing of *Kcnn4* does not induce changes in the expression of ENaC subunits or prostasin (*Prss8*). Transcripts were isolated from airway epithelial cells from wild type (blue dots) or *Kcnn4*$^{-/-}$ (red dots). Values correspond to $2^{-\Delta CT}$ normalized to Cyclophilin; n = 4–5 for each group. Statistical analysis performed using a Rank sum test.
